# Sclerostin

**DOI:** 10.4061/2010/941419

**Published:** 2010-09-29

**Authors:** Stuart L. Silverman

**Affiliations:** Cedars-Sinai, UCLA, OMC Clinical Research Center, 8641 Wilshire Boulevard, Suite 301, Beverly Hills, CA 90211, USA

## Abstract

The striking clinical benefits of intermittent parathyroid hormone in osteoporosis have begun a new era of skeletal anabolic agents. One potential new agent is monoclonal antibody to sclerostin, a potent inhibitor of osteoblastogenesis.

## 1. Introduction

The Wnt signaling pathway demonstrates a complex network of proteins well known for their roles in embryogenesis but also involving normal physiologic processes of bone formation in response to loading and unloading [1]. The Wnt pathway involves a large network of proteins that can regulate the production of Wnt signaling molecules [2]. Several proteins that inhibit Wnt signaling [2] have been described. One such protein is sclerostin which binds low-density lipoprotein receptor-related protein (LRP) and inhibits Wnt signaling. This paper discusses both preclinical and clinical data for sclerostin.

## 2. Sclerostin

Sclerostin which is a potent inhibitor of osteoblastogenesis is a glycoprotein secreted by osteocytes. Sclerostin after secretion by osteocytes travels through osteocyte canaliculi to the bone surface where it binds to coreceptors LRP5 and LRP6 thus preventing colocalization with frizzled protein and Wnt signaling, and thereby reducing osteoblastogenesis and bone formation [3]. 

Loss-of-function mutations in *SOST* are associated with an autosomal-recessive disorder, sclerosteosis, which causes progressive bone overgrowth [4]. A deletion downstream of the *SOST* gene, which results in reduced sclerostin expression, is associated with a milder form of the disease called van Buchem disease [5]. Furthermore, *SOST*-null mice have a high-bone-mass phenotype [6]. 

Sclerostin suppression is required for balanced remodeling in response to PTH [7]. Serum sclerostin levels are significantly higher in postmenopausal women than in premenopausal women with significant negative correlations between free estrogen levels and sclerostin as well as PTh and sclerostin [8].

 The development of a monoclonal antibody to sclerostin that can be administered subcutaneously has allowed scientists to evaluate the effect of sclerostin blockade on bone metabolism and bone mass. Li et al. [9] treated estrogen-deficient osteopenic rats with biweekly subcutaneous treatment with 25 mg/kg of a monoclonal antibody to sclerostin for 5 weeks and restored trabecular bone mass to baseline levels. Surface-based histomorphometry determined that the increase in bone mass resulted from an increase in bone mass at all skeletal envelopes, including cancellous, cortical bone sites, and supervertebral sites. The increase in bone mass and the change in microarchitecture were associated with improved bone strength in both the appendicular and axial skeleton. This data shows that pharmacologic inhibition of sclerostin results in increased bone formation, bone mass, and bone strength in rodents. In a mouse model of colitis, short-term treatment with Scl Ab countered the effects of chronic inflammation on bone loss and resulted in increased bone strength and bone formation [10]. Similarly, in rodent models of fracture healing, Scl Ab treatment resulted in increased callus density and bone strength at fracture sites and accelerated bone repair [11]. This increased bone formation and bone mass by sclerostin antibody was not blunted in ovariectomized rats pretreated with alendronate [12]. Combination of both sclerostin antibody and zoledronic acid resulted in additive effects on bone parameters and bone mass [13]. Treatment with sclerostin inhibitors is not gender specific; treatment increases bone formation in male mice [14].

 Sclerostin inhibition in primates has recently been reported by Ominsky [15]. A humanized sclerostin neutralizing monoclonal antibody (Scl Ab) was administered to gonad-intact female cynomolgus monkeys. Two once monthly subcutaneous injections of Scl Ab were administered at three dose levels (3, 10, and 30 mg/kg) over two months. Scl Ab resulted in dose-dependent increases in bone formation on trabecular, periosteal, endocortical, and intracortical surfaces. Bone density measurement showed significant increases (11–29% compared to vehicle alone) at femoral neck and radial and tibial metaphysis. Additionally significant increases in trabecular thickness and bone strength were seen in the lumbar vertebrae at the highest dose strength. In another study by the same author, sclerostin antibody stimulated bone formation and improved strength of the fracture callus in a primate fibular osteotomy model [16]. Although these studies are short term, they suggest that sclerostin inhibition resulting in increased bone formation may be useful clinically in osteoporosis and fracture healing.

Despite the increases in anabolic activity, no increase in bone resorption as measured by serum CTX was found in the primate study, suggesting that coupling of resorption and formation did not occur consistent with prior results in sclerostin-knockout mice and in oophorectomized rats treated with sclerostin antibody. This may suggest that the mechanism of anabolic action differs from PTH where bone resorption markers are seen within one month of treatment. This may represent direct activation of bone formation (modeling) without activation of bone resorption (bone remodeling).

In humans, antisclerostin antibody results in dose-dependent increases in markers of bone formation in healthy postmenopausal women [17]. 

 The bone-forming effects of the *SOST* antibody resemble in many ways those of high-dose intermittent PTH in rodents. Several studies have reported that sclerostin gene expression and protein levels are reduced in animals treated with daily injections of human parathyroid hormone (hPTH) (1–34). Preclinical studies with a sclerostin inhibitor appear to be somewhat different from those with hPTH (1–34). For example, all skeletal sites respond to anabolic daily PTH treatment; the trabecular bone is most responsive, followed by the endosteal surface and the periosteal surface. In contrast, inhibition of sclerostin also results in significant bone formation at the periosteal surface. Also, studies find the increases in bone formation induced by antisclerostin antibody, unlike PTH, not associated with increases in bone resorption in the aged rodent skeleton. 

Reduced mechanical stimulation leads to disuse osteoporosis, as seen in bedridden patients and in astronauts. Lin et al. recently [18] reported that *SOST*-knockout mice were resistant to mechanical unloading bone loss. In contrast to wild-type mice, Wnt/*β*-catenin signaling was not altered by unloading in *SOST*-knockout mice. The data suggest a potential major role for sclerostin in mediating the bone response to unloading and propose it may be a promising target for preventing disuse osteoporosis [18]. 

 At this time, monoclonal antibody to sclerostin is being considered for early phase 2 clinical trials in postmenopausal women with osteoporosis and in fracture healing. The long-term safety of sclerostin in humans has not been studied. Additional clinical study data is needed to determine if the rapid gain in bone mass is associated with bone of normal strength and architecture and if boney overgrowth occurs at areas such as the carpal tunnel resulting in carpal tunnel syndrome, or around the lumbar spine neural foramen resulting in lumbar radiculopathy or spinal stenosis [19]. 

 In summary, treatments based on inhibition of sclerostin may be a powerful way to restore skeletal bone strength in our patients and may provide more efficacious protection from hip fracture than current therapies as well as potentially improve fracture healing.

## 3. Conclusions

We now have a diverse menu of osteoporosis therapies including both antiresorptive therapy and one anabolic therapy (teriparatide). Current research suggests that in the future we may have multiple different anabolic therapies such as sclerostin. The therapies may have orthopedic benefits in terms of fracture healing and fusions. The future of anabolic therapies looks bright.

##  Disclosure 

The author is a consultant and speaker for Lilly and Amgen.
